# Mitral regurgitation due to caseous calcification of the mitral annulus: two case reports

**DOI:** 10.1186/1757-1626-2-95

**Published:** 2009-01-29

**Authors:** Marcello Marcì, Francesca Lo Jacono

**Affiliations:** 1 Cardiology Department, Azienda Ospedaliera Villa Sofia, P.tta Salerno, 2. Palermo, Italy

## Abstract

Caseous calcification is a rare variant of mitral annular calcification, occurring in about 0.06% of echocardiographic studies performed. It is usually a benign lesion, but it should be differentiated by abscess and tumors. Echocardiography is the most sensitive method to identify caseous calcification which appears typically as a round, calcified mass with an echo-lucent, liquid-like inner part.

## Background

Unlike mitral annular calcification (MAC), that is a common echocardiographic finding, caseous calcification is a rare variant, occurring in about 0.6% of patient with annular calcification [1-3]. Patient 1.

An 82-year-old woman, with history of hypertension, was admitted to our department for paroxystic atrial fibrillation, which was successfully treated with intravenous Amiodarone. Physical examination revealed a systolic murmur of 2–3/6 L grade. Besides laboratory examinations were substantially normal.

**Figure 1:**
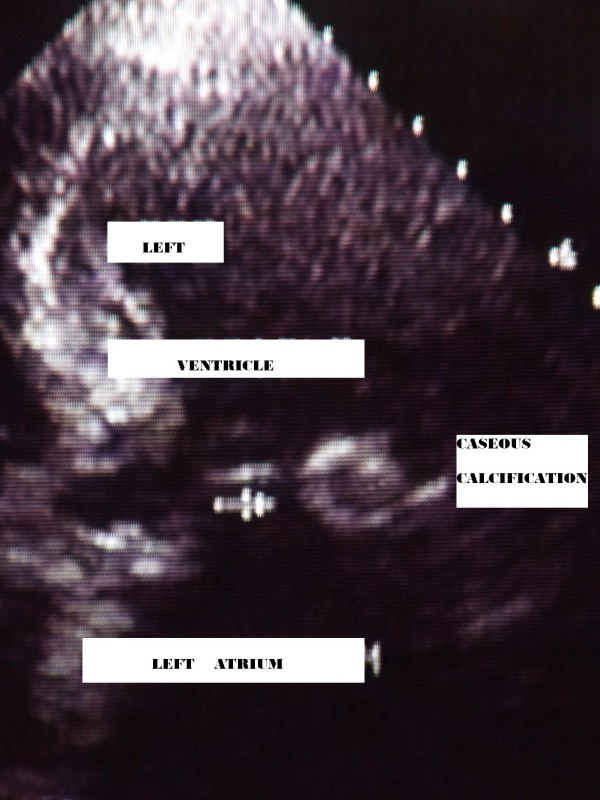
**apical four chamber view**.

Echocardiogram demonstrated mild left atrial enlargement (diameter = 42 mm, area = 22 cm^2^), therefore left ventricle was hypertrophic with an ejection fraction of about 60%. Additionally in the posterior site of mitral annulus, at the base of corresponding leaflet, was a round cystic mass of about 2 cm in diameter, with a calcified capsule and an echo-lucent core (Fig. 1). The lesion was consistent with caseous calcification of mitral annulus.

Colour-Doppler examination ruled out shunt between the cyst and the cardiac chambers, moreover colour flow mapping revealed a moderate mitral insufficiency without mitral stenosis. Transesophageal echocardiogram was refused by the patient.

Since there was not dyspnoea, nor left ventricular dilatation, conservative treatment and follow up was considered sufficient.

**Figure 2:**
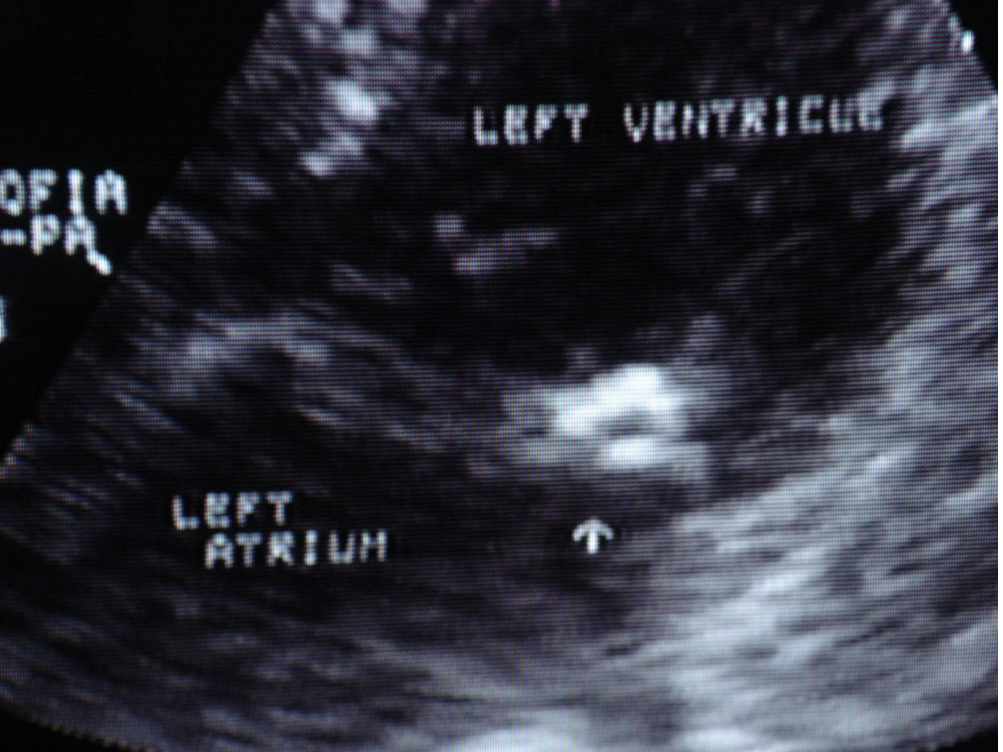
**apical four chamber view**.

Patient 2. A 71-years-old woman was referred to our institution because of anterior acute myocardial infarction, about 6 hours after the onset of symptoms. Electrocardiogram showed sinusal rhythm and ST elevation in precordial V1 → V4 leads. She did not complain dyspnea (Killip class 1). Coronary arteriogram showed a sub-occlusion (TIMI 1–2) of mid-portion of the left anterior descending artery, that was successfully treated with percutaneous coronary intervention with stent implantation. On auscultation a 3/6 grade systolic murmur was heard at the lower left sternal border. Echocardiogram evidenced an hypertrophic left ventricle of normal dimension, with apical akinesis, ejection fraction was 48%. Moreover posterior mitral annulus appeared calcified with a rounded, vacuolated mass of 1 cm in diameter (Fig. 2). Colour-Doppler examination demonstrated mild aortic insufficiency and moderate mitral regurgitation. Transesophageal echocardiogram confirmed that mitral posterior leaflet was thickened and partially calcified, otherwise chordate tendineae appeared normal but the posterior valvular ring and the base of leaflet was deformed by a cystic mass consistent with caseous calcification.

## Comments

MAC is a common finding, especially in the elderly and in the end-stage renal failure [1-5]. Calcification begins between the posterior atrio-ventricular groove and the base of posterior mitral leaflet, although in very elderly subjects it can involve the entire annulus. Otherwise caseous calcification represents a rare evolution of a calcified mitral ring, due to caseous transformation of the inner material.

Histological examination of the inner fluid usually reveals an amorphous, basophilic content, composed by a blend of calcium and cholesterol, surrounded by a inflammatory cells, mainly macrophages [3-5]. Echocardiography has demonstrated to be the most reliable method to diagnose this lesion. Almost invariably, caseous calcification appears as an echo-dense, round, smooth mass with a liquid core, surrounded by a calcified envelop, as a rule it is localized into the posterior mitral annulus [1-3,6]. Further exams, such as transesophageal echocardiography, computed tomography and magnetic resonance, are seldom necessary to differentiate caseous calcification from tumour or abscess [3]. In fact the latter condition usually causes fever and embolic events. Besides caseous calcification has a more calcified envelope than infected mitral ring [3].

Caseous calcification is a rare, accidental echocardiographic finding, occurring in about 0, 06% of all echocardiographic studies [3-6], whereas it has a higher prevalence in a necropsy series [4]. This discrepancy could be explained by the fact that this condition could not be recognised by cardiologists that are unfamiliar with this unusual lesion. It is of interest that this pathological condition is mentioned as sterile caseous abscess of mitral ring in a widely diffuse textbook of echocardiography [7] and it is neglected by a recent review of echogenic structures [8].

Caseous calcification is a benign condition, which only rarely may determine significant valvular stenosis or insufficiency, requiring surgical treatment [3-6,9]. As a rule conservative management is indicated in most of the cases [1-3].

## Competing interests

The authors declare that they have no competing interests.

## Consent

The authors state that informed written consents were obtained from both the patients for publication of manuscript and figures.

## Authors' contributions

Both Authors, MM and FLJ, have contributed to collect clinical data and have performed ecohocardiograms of the second patient. MM has performed clinical examination and echocardiogram of the first patient moreover he has found bibliography. The manuscript was made and revised by both the Authors.

## References

[B1] Novaro GM Griffin BP Hammer DF Caseous calcification of the mitral annulus: an underappreciated variant Heart; 2004 90 4 388 15020511 10.1136/hrt.2003.023010 PMC1768159

[B2] Arora H Madan P Simpson L Stainback RF Caseous calcification of the mitral annulus Tex Heart Inst J 2008 35 2 211 213 18612497 PMC2435443

[B3] Harpaz D Auerbach I Vered Z Motro M Tobar A Rosenblatt S Caseous calcification of the mitral annulus: a neglected, unrecognized diagnosis J Am Soc Echocardiogr 2001 14 825 831 10.1067/mje.2001.111877 11490332

[B4] Pomerance A Pathological and clinical study of calcification of the mitral valve ring J Clin Pathol 1970 23 354 361 5430424 10.1136/jcp.23.4.354 PMC476757

[B5] Sheppard M Davies M Practical cardiovascular pathology 1998 New York. Oxford University Press 86

[B6] Kronzon I Winer HE Cohen ML Sterile, caseous mitral annular abscess J Am Coll Cardiol 1983 2 186 190 6853912 10.1016/s0735-1097(83)80391-x

[B7] Feigenbaum H Echocardiography 1994 5 Lea & Febiger 329

[B8] Zuber M Oechslin E Jenni R Echogenic structure in the left atrioventricular groove: diagnostic pitfalls J Am Soc Echocardiogr 1998 11 381 386 10.1016/S0894-7317(98)70107-5 9571589

[B9] Minardi G Manzara C Pulignano G Pino PG Pavaci H Sordi M Musumeci F Caseous calcification of the mitral annulus with mitral regurgitation and impairment of functional capacity: a case report Journal of Medical Case Reports 2008 2 205 209 18549500 10.1186/1752-1947-2-205 PMC2440756

